# NEITHER study: NICE versus EAU inconsistent triage, expenses and reliability in non–high‐risk non‐muscle invasive bladder cancer surveillance

**DOI:** 10.1002/bco2.70283

**Published:** 2026-10-14

**Authors:** Youssef Ibrahim, Luis Ribeiro, Michelle Heelan, Martina Spazzapam, Findlay Macaskill, Kiyara Fernando, Adham Ahmed, Ahmed Hussien, Mohammed Gad, Soumitra Chakravarti, Sara Mokbel, Rajesh Nair, Ramesh Thurairaja, Muhammad Shamim Khan, Yasmin Abughanem, Elsie Mensah

**Affiliations:** ^1^ Guy's Hospital London UK; ^2^ St George's Hospital London UK; ^3^ Centre for Transplantation, Urology and Kidney Disease King's College London UK; ^4^ Addenbrooke's Hospital Cambridge UK

**Keywords:** cost‐effectiveness, EAU guidelines, NICE guidelines, NMIBC, non–muscle‐invasive bladder cancer, recurrence, risk stratification, surveillance

## Abstract

**Objective:**

This study aimed to quantify discrepancies in non–high‐risk non–muscle‐invasive bladder cancer (NMIBC) stratification between the National Institute for Health and Care Excellence (NICE) and the European Association of Urology (EAU) guidelines, compare discriminative performance for predicting recurrence, estimate potentially missed recurrences under shorter surveillance and calculate per‐patient surveillance costs.

**Patients and methods:**

This is a retrospective cohort study of patients undergoing transurethral resection of bladder tumour (TURBT) at a UK tertiary centre (2018–2020). Patients with low‐ or intermediate‐risk NMIBC who completed 5‐year follow‐up or experienced recurrence were included. Each patient was stratified according to both NICE and EAU (WHO 1973) guidelines. Outcomes included recurrence rate, discriminative accuracy (Harrell's C‐index), proportion of recurrences occurring beyond NICE discharge timepoints and surveillance cost based on NHS reference prices.

**Results:**

Of the 158 eligible patients (78% male; mean age 68 years), 40% were assigned different risk categories depending on guideline applied. NICE classified significantly more patients as low risk than EAU (31.1% vs. 12.7%; *p* < 0.001). Overall, 5‐year recurrence was 48.7%, with no significant difference in recurrence rates between corresponding risk groups under each guideline. NICE demonstrated superior discrimination (C‐index 0.605 vs. 0.511; *p* = 0.006). Among NICE low‐risk patients, 45% of recurrences occurred between Years 2 and 5—beyond the recommended 1‐year discharge point. Mean per‐patient surveillance cost was £1412 under NICE versus £3525 under EAU, representing a 60% reduction.

**Conclusions:**

NICE can reduce cost with comparable discrimination efficiency to EAU guidelines, but risks missed recurrences; a hybrid NICE–EAU approach may offer the optimal balance.

## 1 INTRODUCTION

Non–muscle‐invasive bladder cancer (NMIBC) is among the most expensive malignancies to manage on a per‐patient basis, largely owing to the need for repeated endoscopic surveillance and treatment of recurrences.^1^ However, NMIBC represents a clinical spectrum with heterogeneous risks of recurrence and progression, making risk‐adapted management essential to direct resources where they are most needed. Therefore, several prognostic models based on routinely assessed clinical and pathological factors are available.^2^, ^3^, ^4^, ^5^

The European Association of Urology (EAU) and the National Institute for Health and Care Excellence (NICE) guidelines both provide risk stratification frameworks, yet their criteria differ substantially—particularly for low‐ and intermediate‐risk disease, where supporting evidence is limited and recommendations rely heavily on expert consensus. The adoption of NMIBC guidelines varies considerably across the United Kingdom, resulting in significant discrepancies in patient management and surveillance recommendations.^6^

These differences have practical consequences. The risk category assigned to a patient determines not only the intensity and duration of surveillance but also the eligibility for adjuvant intravesical therapy. Understratification may lead to inadequate follow‐up and missed recurrences; overstratification may expose patients to unnecessary procedures and costs. Understanding how guideline selection affects real‐world practice is therefore critical.

### 1.1 Stratification model

Stratification models categorize NMIBC by risk, allowing a personalized treatment and surveillance plan. The European Organization of Research and Treatment of Cancer (EORTC) 2006 scoring model identified significant prognostic factors to quantify risk of recurrence and progression.^5^ Across various guidelines, the most important prognostic factors for predicting progression are tumour stage, grade and the presence of carcinoma in situ (CIS). The presence of T1, G3 and/or CIS characterizes high‐risk NMIBC across all guidelines. In contrast, non–high‐risk NMIBC represents a more heterogeneous population, characterized by a lower risk of progression (<1% at 1 year) but a high risk of recurrence.^5^ Subcategorization of non–high‐risk NMIBC into low‐ and intermediate‐risk groups incorporates factors such as tumour number and size and prior recurrence or recurrence rate.^3^, ^5^ Utilization of these factors varies between guidelines, resulting in substantial differences in the risk category assigned to patients (low or intermediate) and thus differences in surveillance schedules and related costs.

In 2021, the European Association of Urology introduced a revised risk stratification model for NMIBC that incorporates both the WHO 1973 and WHO 2004/2022 grading systems in parallel.^7^ This approach provides separate risk categorization for each grading system, allowing flexibility for centres using either classification. When both grading systems are available, the panel recommends prioritizing the WHO 1973 system due to its stronger prognostic performance. The divergence in risk stratification between WHO 1973 and 2004/22 is particularly evident in low‐ and intermediate‐risk NMIBC cases.^7^ However, evidence suggests that a hybrid approach that integrates both grading systems offers superior prognostic accuracy compared to either system alone.^8^, ^9^

### 1.2 Surveillance protocol

Surveillance aims to detect recurrence at a stage amenable to curative intervention. All guidelines recommend lifelong follow‐up for high‐risk NMIBC. For lower‐risk disease, recommendations diverge substantially. NICE recommends discharge of low‐risk patients after 1 year (two cystoscopies) and intermediate‐risk patients after 5 years (six cystoscopies). EAU recommends surveillance for 5 years in low‐risk disease (six cystoscopies) and 10 years in intermediate‐risk disease (13 cystoscopies). These differences have major implications for procedural burden and cost.^4^, ^5^

### 1.3 International guidelines comparison

The 2024 AUA/SUO guideline differs from both NICE and EAU in its risk stratification and surveillance of NMIBC, despite also using a three‐tier classification system.^10^ Like NICE, it assigns risk using predefined clinicopathological criteria without additional risk variables; however, it applies the two‐tier 2004/2022 WHO grading system and classifies low‐grade T1 disease as intermediate risk. Surveillance is risk‐adapted, but the recommended intervals allow considerable flexibility for personalized, shared decision‐making.^10^ Although clinically pragmatic, the absence of a prescriptive surveillance schedule complicates estimation of exact costs and the clinical and economic consequences of missed follow‐up.

## 2 AIM OF THE STUDY

The impact of various risk stratification models and surveillance protocols on patients and healthcare systems is substantial. Understratification may lead to undertreatment and early discharge, increasing the risk of missed recurrence. Conversely, overstratification can result in overtreatment, exposing patients to unnecessary cystoscopies with physical and psychological burden, while increasing costs without added benefit. Guideline recommendations must therefore balance service capacity and cost‐effectiveness without compromising patient safety.^9^

In this study, we evaluated discrepancies in non–high‐risk NMIBC stratification between EAU and NICE, assessed the impact of risk reclassification on recurrence rates, estimated potentially missed recurrences with shorter surveillance and calculated per‐patient cost of each surveillance protocol.

## 3 METHODS

This is a retrospective comparative cohort study in keeping with STROBE guideline, comparing performance of NICE versus EAU guidelines ‘stratification model and surveillance protocol for NMIBC’.^11^

### 3.1 Study population

All patients who underwent Transurethral Resection of the Bladder Tumours (TURBT) between 2018 and 2020 at our tertiary centre were identified. Patients with low and intermediate risk NMIBC that had either completed the 5‐year surveillance period or recurred at any timepoint were included in the study. Patients with incomplete resection, final benign pathology and history of upper tract urothelial carcinoma, high‐risk NMIBC or MIBC were excluded. Patients with final pathology of NMIBC were risk stratified based on NICE and EAU guidance. The EAU 2021 online risk calculator was used to identify the risk‐category of each patient. Risk stratification according to WHO‐1973 grading system was utilized as control group, as recommended by EAU to be better prognosticator.^4^, ^7^ Discrepancies in risk stratification between the two guidelines were quantified within same patient cohort minimizing bias.

### 3.2 Primary and secondary outcomes

The co‐primary outcome was recurrence and progression rates; cystoscopy‐based identification of recurrence was used as the definition for recurrence. Progression was defined as histological diagnosis of MIBC on resection of recurrence.

Secondary outcomes included time‐to‐recurrence, the proportion of recurrences that would have been missed by the shorter surveillance protocol, EORTC recurrence and progression scores and the surveillance costs according to guideline applied; surveillance costs were estimated using the 2025–2026 NHS reference cost of £291 per flexible cystoscopy.^12^ Total surveillance costs were calculated by multiplying the number of cystoscopies recommended by each guideline (NICE: low‐risk, 2; intermediate‐risk, 6; EAU: low‐risk, 6; intermediate‐risk, 13) by the number of patients assigned to each risk category.

### 3.3 Statistical methods

The study size was determined by the number of eligible patients available during the study period. Continuous variables were summarized using means (range), and categorical variables as counts and percentages. Categorical comparisons were performed using Fisher's exact test and continuous variables using the Mann–Whitney U test where appropriate. Time‐to‐recurrence was analysed using the Cox regression.

Model discrimination at 5 years was evaluated using Harrell's bias‐corrected concordance index (c‐index), which quantifies the probability that, for a randomly selected pair of patients, the individual who experiences recurrence earlier is assigned a higher predicted risk by the model.

All tests were two‐sided, with *p* < 0.05 considered statistically significant. Analyses were performed using IBM SPSS Statistics version 29 (IBM Corp., Armonk, NY, USA).

### 3.4 Ethical approval and patients' consents

This study was based on a retrospective review of routinely collected clinical data obtained from patient medical records. The UK Medical Research Council (MRC) decision tool was applied to assess the need for NHS Research Ethics Committee approval, which confirmed that the project met the criteria for service evaluation and did not require formal ethical review. As the analysis was conducted using fully anonymised data and did not alter patient management, individual informed consent was not required. Data were managed in accordance with local governance policies and in line with the principles of the Declaration of Helsinki.

## 4 RESULTS

### 4.1 Study population

Out of the total 320 patients who underwent TURBT between 2018 and 2020, 246 patients were diagnosed with NMIBC. Figure 1 illustrates that inclusion and exclusion processes of these 158 patients were included in the study. A total of 158 low/intermediate risk NMIBC patients met the inclusion criteria for the study, of which 123 were men (78%), with mean age of 68 years (range 24–95). Patients' demographics and tumour characteristics are illustrated in Table 1.

**FIGURE 1:**
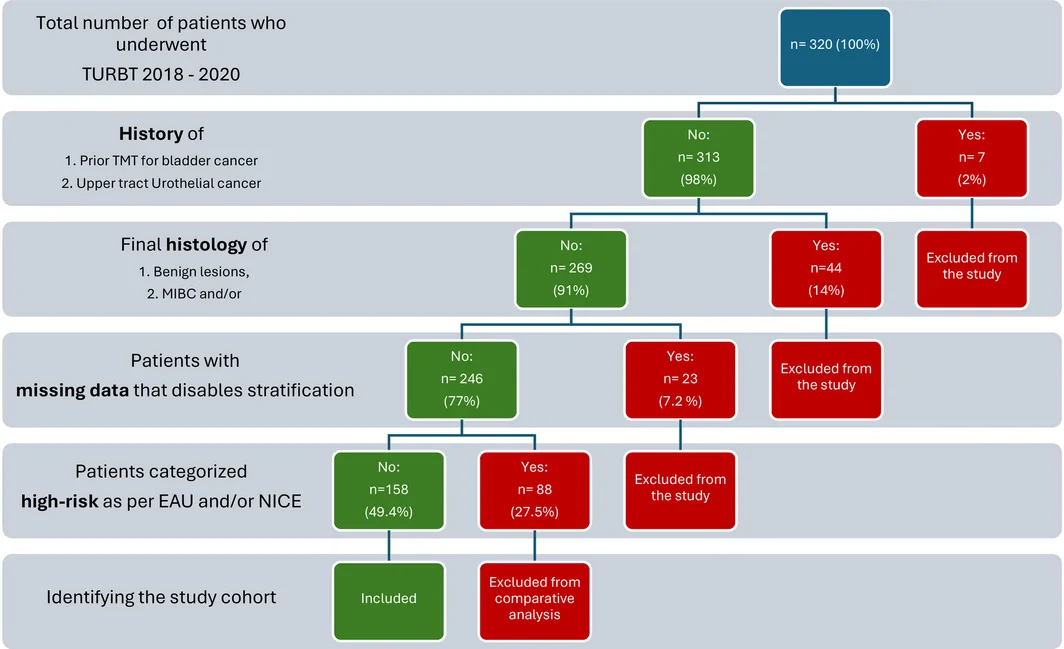
Outlines the inclusion and exclusion process leading to the final study cohort.

**TABLE 1:** Demographics of study population.

Patient characteristic	Number (%)
Age	
≤ 70 years old	76 (48%)
> 70 years old	82 (52%)
Gender	
Male	123 (78%)
Female	35 (22%)
SI‐MMC treatment	
Yes	28 (18%)
No	130 (82%)
Prior recurrence	
Primary	76 (48%)
Rec, ≤ 1 rec/year	37 (23%)
Rec, > 1 rec/year	45 (29%)
Number of tumours	
1	91 (58%)
2–7	53 (33%)
≥ 8	14 (9%)
Tumour size	
< 3 cm	136 (86%)
≥ 3 cm	22 (14%)
Grade	
LG/G1	44 (28%)
LG/G2	81 (51%)
HG/G2	33 (21%)

### 4.2 Variability of risk stratification and recurrence rate

Patients were risk‐stratified according to both NICE and EAU guidelines. Of the cohort, 95 (60%) had similar risk stratification according to both guidelines. The proportion of patients classified as low risk was significantly higher according to NICE guidelines, 49 of the 158 patients (31.1%) compared with only 20 (12.7%) applying EAU guidelines (absolute difference 18.4%, 95% CI 9.5%–27.2%, *p* < 0.001). (Table 2).

**TABLE 2:** Comparison of low and intermediate risk categories according to NICE versus EAU.

Risk group	Variable	NICE	EAU (WHO 1973)	*P*‐value
Low	Number (*t*: 158) *n*, %	49, 31.1%	20, 12.7%	< 0.001*
	Recurrence rate *n*, %	11, 22.4%	7, 35.0%	0.37
	EORTC recurrence score Mean (range)	1.3 (0–4)	1.1 (0–4)	N.A.
	EORTC progression score Mean (range)	0.7 (0–3)	0.7 (0–3)	N.A.
Intermediate	Number (*t*: 158) *n*, %	109, 68.9%	138, 87.3%	< 0.001*
	Recurrence rate *n*, %	66, 60.6%	70, 50.7%	0.16
	EORTC recurrence score Mean (range)	5.9 (1–14)	4.8 (1–14)	N.A.
	EORTC progression score Mean (range)	4.0 (0–9)	3.2 (0–9)	N.A.

Overall, 5‐year recurrence rate within the non–high‐risk group of patients was 48.7% (*n* = 77) in our study. The specific recurrence rate for various subgroups is illustrated in Table 2. The difference in recurrence rate was not statistically significant between low‐risk patient groups according to both guidelines, as well as for intermediate‐risk patient groups. The percentage of recurrences missed due to early discharge for low‐risk patients as per NICE was 45% (*n* = 5/11).

Applying the EAU 2021 scoring model, when both the WHO‐1973 and WHO‐2004/2022 were inserted, discordant stratification resulted in 48 patients (30%) falling into two different risk categories (*p* = 0.018).^9^ There was no difference between WHO 2004/2022 and WHO 1973 recurrence rates among low and intermediate risk patients (*p* = 0.68 and 0.54, respectively).

### 4.3 Appropriateness of risk classification

Comparing optimism corrected Cox C‐index (C‐index) between the two guidelines to assess discriminative accuracy for predicting tumour recurrence, none of the tested guidelines had good or excellent discriminative accuracy (C‐index>0.7). The NICE classification demonstrated higher discriminative performance with a C‐index of 0.605 compared to 0.511 for EAU, representing an improvement of 0.094 over the EAU (95% CI 0.035–0.130, *p* = 0.006).

The Net Reclassification Index (NRI) was used to assess accuracy of NICE versus EAU risk‐prediction by differentiating those patients with and without recurrences. Of the 77 patients who had recurrence, 69 had the concordant risk category according to both guidelines, and 8 were different; 2 cases were appropriately reclassified to a higher‐risk group, while 6 were inappropriately downgraded using NICE guidelines compared to EAU. In contrast, among nonrecurrent patients, 26 were appropriately downgraded and 3 were upgraded. This resulted in an NRI 0.232, indicating a 23.2% improvement in appropriate classification compared with EAU (Table 3).

**TABLE 3:** Net reclassification index of NICE guidelines versus EAU‐WHO 1973 differentiating recurrent cases.

Reclassification table (converting EAU to NICE)	Total	Reclassified up (low risk‐ > Int. risk)	Reclassified down (int. risk ‐ > low risk)	Unchanged
Cases (with recurrence)	77	2	6	69
Controls (no recurrence)	81	3	26	52

*Note*: NRI results: NRI for cases: −0.0519 NRI for controls: 0.2840 Total NRI: 0.2320.

### 4.4 Surveillance costs depending on guidelines applied

Using the NICE stratification model our patient cohort would have undergone a total of 752 flexible cystoscopies compared to 1914 using the EAU risk stratification model. This results in an average of 4.9 cystoscopies per patient using the NICE model compared to 12.1 cystoscopies per patient using the EAU model.

The overall procedural cost of surveillance would be £218 832 using the NICE model compared to £556 974 using the EAU model, following NHS England schedule costs.^13^ The mean cost per patient would be £1411.82 using NICE compared to £3525.15 using the EAU model. Overall, the NICE guideline would result in a saving of 65% when compared to the EAU guideline (Table 4).

**TABLE 4:** Comparison of total surveillance costs and average cost per patient, according to different guidelines.

Metrics	NICE (1 year)	EAU
Low risk (*n*/total)	49/158	20/158
Total cystoscopies	752	1914
Avg cystoscopies/patient	4.9	12.1
Total cost	£218832.00	£556974.00
Avg cost/patient	£1411.82	£3525.15

## 5 DISCUSSION

Even minor differences in definitions, classification criteria and management recommendations between guidelines translate into clinically meaningful variation in real‐world practice. While international guidelines aim to define an evidence‐based gold standard, implementation constraints such as service capacity, resource allocation and cost can impact adherence, as well as ease of use.^2^, ^6^ Our study provides a direct, real‐world comparison of NICE and EAU recommendations within the same NMIBC cohort, demonstrating that guideline selection alone can substantially alter patient management and expenditure burden.

### 5.1 Real‐world variability

Although clinicians and centres have the flexibility to choose guidelines that suit their settings, the resulting variation in practice and its impact on patients and healthcare systems should not be underestimated. Differences in non–high‐risk NMIBC risk stratification between the NICE and EAU guidelines arise from the variable weighting of tumour size, multiplicity, recurrence status, recurrence rate and patient age. In our cohort, 40% (63/158) of non–high‐risk NMIBC patients were assigned to different risk categories depending on the guideline applied which is statistically significant (*p* < 0.001). Meanwhile, the variation in stratification according to EAU guidelines using WHO‐2004 grading versus WHO‐1973 grading affected 30% of patients which is also statistically significant (*p* = 0.018). These findings highlight substantial variability in practice, both between and within guideline recommendations. Sylvester et al. applied the EAU guidelines using WHO 2004/2022 versus WHO 1973 that led to substantial reclassification across all risk categories: Low‐risk increased by 95% and intermediate‐risk decreased by 53%, while changes in high‐risk were (+16%) when WHO‐2004/2022 grading system is applied compared to standard WHO‐1973. Notably, the greatest discordance occurs within the non–high‐risk groups.^1^

### 5.2 Stratification model efficiency

Recurrence rates did not differ significantly between risk groups, indicating no meaningful increase in recurrence when applying one guideline over the other. No patients have progressed within our series. EORTC progression and recurrence risk scores were comparable between risk groups irrespective of stratification model applied. In Sylvester study, although no formal comparison was performed, progression rates appear comparable across both WHO grading systems, despite marked redistribution between low‐ and intermediate‐risk groups.^1^

Overall, the ability of either guideline to discriminate between low‐ and intermediate‐risk disease was limited with C‐index <0.7. Nevertheless, NICE demonstrated statistically significant improvement in C‐index (0.04–0.09) compared to both EAU classification systems. Sylvester et al. calculated higher C‐index for EAU guidelines in their series. However, including high‐risk NMIBC would significantly improve overall discrimination, masking the poor discriminatory ability between low and intermediate risk disease.^1^

The superiority of NICE stratification model might be attributed to adoption of 4‐tier histopathological grading system (G1, LG/G2, HG/G2 and G3). Studies evaluating progression risk have shown that three‐ or four‐tier models offer superior prognostic performance compared to the individual WHO grading systems used within EAU. While EAU guidelines aim to provide flexibility across healthcare systems, national guidance should align with local practice. Given that most UK pathology reports use a four‐tier system, its adoption would be logical, offering improved prognostic accuracy alongside the cost‐effectiveness demonstrated in this study.^9^

### 5.3 Surveillance duration and safety

The higher proportion of patients categorized low risk within NICE means that more patients will have less intense surveillance without added risk; this is not to be underestimated as patients related outcome studies showed increased discomfort and anxiety with endoscopic surveillance for NMIBC.^14^ Nevertheless, intermediate risk‐NMIBC would be offered intravesical BCG which comes with its own morbidity as well as healthcare system challenges with global shortages.^15^

However, deintensification comes with it is own risk of missed recurrence. In our series, 45.5% of recurrences in NICE low‐risk patients occurred between Years 2 and 5 of surveillance, which would have been missed or diagnosed late if patients were discharged according to NICE recommendations. Although the overall risk of progression remains low, Sylvester et al. demonstrated that progression risk in low‐risk NMIBC is higher during years 2–5 compared with the first year, irrespective of the WHO grading system. These findings highlight the potential risk of missed clinically relevant recurrences associated with early discharge under NICE guidance.^1^

### 5.4 Costs' impact

While differences in guidelines' categorization ability are modest, costs are significantly higher applying the EAU guidelines compared to NICE, irrespective of WHO‐grading system utilized to risk stratify. NICE cost‐reduction was derived from its stratification model and the shorter surveillance duration. The number of patients categorized as low‐risk per NICE was 145% more than those given same risk‐category according to EAU‐1973. However, the shorter NICE surveillance schedule would have resulted in nearly half of recurrences in the low‐risk group being missed following discharge at 1 year in our cohort. Long‐term data further challenge early discharge strategies. In a population‐based cohort with 25‐year follow‐up, patients with non–high‐risk NMIBC demonstrated persistent risk of progression and cancer‐specific mortality, with T‐stage progression occurring at a median of 49 months and most cancer‐related deaths occurring between 5 and 9 years after diagnosis. These findings indicate that clinically relevant events occur well beyond the early surveillance period, highlighting the potential risk of missed or delayed diagnosis associated with abbreviated follow‐up protocols such as those recommended by NICE.

### 5.5 Need for novel/updated national guidance

Our findings highlight the relative strengths and limitations of both guidelines, widely adopted in the United Kingdom, when applied to a real‐world cohort, suggesting that neither is ideal for routine practice. The EAU risk stratification model is more complex and less user‐friendly and results in substantial up‐classification between low‐ and intermediate‐risk groups, with a corresponding increase in cost. In contrast, NICE facilitates earlier discharge but at the expense of a higher risk of missed or lately diagnosed recurrence. A hybrid approach, incorporating NICE risk stratification with extended surveillance schedules, may reduce the risk of missed recurrences while preserving cost savings compared with EAU. The cost of a proposed extended surveillance for low and intermediate NICE‐risk categories up to 5 and 10 years, respectively, was calculated. The total cost would be 420 204 pounds for an additional 741 cystoscopies. This would increase the average cost/patient to 2659.5 pounds. This would still reduce costs by 25% compared with EAU.

The role of biomarkers and urinary comprehensive genomic testing could inform risk stratification and more personalized surveillance of NMIBC according to their individual risk.^16^

## 6 LIMITATIONS OF THE STUDY

Limitations of this study include its retrospective, single‐centre design, which may limit the generalizability of the findings. The cost analysis did not account for downstream costs associated with delayed recurrence detection, subsequent interventions or variation in real‐world adherence to surveillance schedules and therefore may have underestimated the true economic impact of each surveillance strategy. Recurrence was used as the primary outcome, although progression is arguably a more clinically meaningful endpoint for risk stratification. However, given the low progression rates among non–high‐risk NMIBC, progression would have provided limited statistical power and reduced the interpretability of the analysis. Furthermore, although less intensive surveillance may delay the detection of some low‐grade recurrences, the clinical significance of such delays is likely limited, as many low‐grade recurrences have minimal progression potential and may not require immediate intervention. Accordingly, recurrence rates alone may overestimate the clinical consequences of reduced surveillance intensity.

## 7 CONCLUSION

NICE can reduce cost with comparable discrimination efficiency to EAU guidelines, but risks missed recurrences; a hybrid NICE–EAU approach may offer the optimal balance.

## AUTHOR CONTRIBUTIONS

**Youssef Ibrahim:** Conceptualisation; data collection; writing—original draft. **Luis Ribeiro:** Statistical analysis. **Michelle Heelan:** Data collection. **Martina Spazzapam** and **Kiyara Fernando:** Writing—original draft. **Findly Makascill** and **Adham Ahmed:** Writing—review and editing. **Ahmed Hussien, Mohammed Gad, Soumitra Chakravarti,** and **Sara Mokbel:** Data collection. **Rajesh Nair, Ramesh Thurairaja, Muhammed Shamim Khan, Yasmin Abughanem,** and **Elsie Mensah:** Supervision; writing—review and editing.

## CONSENT

As the analysis was conducted using fully anonymised data and did not alter patient management, individual informed consent was not required. Data were managed in accordance with local governance policies and in line with the principles of the Declaration of Helsinki.

## CONFLICT OF INTEREST STATEMENT

The authors declare no conflicts of interest.

## ETHICS STATEMENT

This study was based on a retrospective review of routinely collected clinical data obtained from patient medical records. The UK Medical Research Council (MRC) decision tool was applied to assess the need for NHS Research Ethics Committee approval, which confirmed that the project met the criteria for service evaluation and did not require formal ethical review.

## AI USAGE DECLARATION

Artificial intelligence (ChatGPT, OpenAI, USA) was used for grammar check and language refining. All authors reviewed and take full responsibility for the final content.

## Data Availability

The data that support the findings of this study are available on request from the corresponding author. The data are not publicly available due to privacy or ethical restrictions.
